# Multimorbidity patterns and their relationship to mortality in the US older adult population

**DOI:** 10.1371/journal.pone.0245053

**Published:** 2021-01-20

**Authors:** D. Diane Zheng, David A. Loewenstein, Sharon L. Christ, Daniel J. Feaster, Byron L. Lam, Kathryn E. McCollister, Rosie E. Curiel-Cid, David J. Lee

**Affiliations:** 1 Department of Psychiatry and Behavioral Science, Center for Cognitive Neurosciences & Aging, University of Miami Miller School of Medicine, Miami, Florida, United States of America; 2 Department of Public Health Sciences, University of Miami Miller School of Medicine, Miami, Florida, United States of America; 3 Department of Human Development and Family Studies and Statistics, Purdue University, West Lafayette, Indiana, United States of America; 4 Bascom Palmer Eye Institute, University of Miami Miller School of Medicine, Miami, Florida, United States of America; Indiana University Purdue University at Indianapolis, UNITED STATES

## Abstract

**Background:**

Understanding patterns of multimorbidity in the US older adult population and their relationship with mortality is important for reducing healthcare utilization and improving health. Previous investigations measured multimorbidity as counts of conditions rather than specific combination of conditions.

**Methods:**

This cross-sectional study with longitudinal mortality follow-up employed latent class analysis (LCA) to develop clinically meaningful subgroups of participants aged 50 and older with different combinations of 13 chronic conditions from the National Health Interview Survey 2002–2014. Mortality linkage with National Death Index was performed through December 2015 for 166,126 participants. Survival analyses were conducted to assess the relationships between LCA classes and all-cause mortality and cause specific mortalities.

**Results:**

LCA identified five multimorbidity groups with primary characteristics: “healthy” (51.5%), “age-associated chronic conditions” (33.6%), “respiratory conditions” (7.3%), “cognitively impaired” (4.3%) and “complex cardiometabolic” (3.2%). Covariate-adjusted survival analysis indicated “complex cardiometabolic” class had the highest mortality with a Hazard Ratio (HR) of 5.30, 99.5% CI [4.52, 6.22]; followed by “cognitively impaired” class (3.34 [2.93, 3.81]); “respiratory condition” class (2.14 [1.87, 2.46]); and “age-associated chronic conditions” class (1.81 [1.66, 1.98]). Patterns of multimorbidity classes were strongly associated with the primary underlying cause of death. The “cognitively impaired” class reported similar number of conditions compared to the “respiratory condition” class but had significantly higher mortality (3.8 vs 3.7 conditions, HR = 1.56 [1.32, 1.85]).

**Conclusion:**

We demonstrated that LCA method is effective in classifying clinically meaningful multimorbidity subgroup. Specific combinations of conditions including cognitive impairment and depressive symptoms have a substantial detrimental impact on the mortality of older adults. The numbers of chronic conditions experienced by older adults is not always proportional to mortality risk. Our findings provide valuable information for identifying high risk older adults with multimorbidity to facilitate early intervention to treat chronic conditions and reduce mortality.

## Introduction

Advances in modern medicine have substantially increased life expectancy, as a result, more people are living into old age and developing chronic conditions. Multimorbidity is the presence of two or more chronic health conditions in an individual [1]. In the United States, the number of older adults living with multimorbidity has increased rapidly [2]. According to a report by RAND, 81% of Americans 65 years and older and 50% aged 45 to 65 years had multiple chronic conditions [3]. In a study of Medicare beneficiaries, 64% of participants had two or more conditions and 24% had four or more [4]. The prevalence of multi-morbidity also has increased in other regions of the world over the past 20 years, and is anticipated to continue rising [2,5,6]. Multi-morbidity is associated with poor daily functioning, increased psychological distress and disability, higher medical utilization and reduced quality of life [4,7,8]. The prevalence of multi-morbidity increases with age [9]. Approximately 71% of US total health care spending is for the treatment of patients with multi-morbidity [10].

Studies have linked multimorbidity to increased risk of mortality [11], but the results are inconclusive [11–17] and most studies had relatively small samples. Previous studies have measured multimorbidity as a cumulative count of conditions or a combination of count and severity of diseases (i.e., Charleston comorbidity index) rather than a qualitative combination of conditions. Multimorbidity increases the risk of adverse consequences to the physiological system from potential interactions between the morbidities and disease treatment. Clusters of conditions may have a synergistic effect on disability or mortality. Investigating chronic condition combination types is crucial in understanding the effect of multi-morbidity on health and mortality.

Latent class analysis (LCA) is a data driven statistical approach that classifies individuals into homogeneous subgroups based on their pattern of response across several observed variables such that individuals within a group are more similar than individuals between groups. Therefore, LCA can be effectively used to determine distinct multimorbidity combination types [18]. Although LCA has been performed to evaluate multimorbidity in various populations [19–24], to our knowledge, LCA has not been applied in examining the multimorbidity combination types and their relationship to mortality in a national representative sample of community residing US older adults. The purpose of this study was to employ LCA techniques to identify patterns of multimorbidity in the US 50 years and older population and evaluate their relationships with all-cause and cause-specific mortalities by utilizing the recent linkage between the National Health Interview Survey (NHIS) and the National Death Index (NDI) [25].

## Methods

### Data source

This cross-sectional study with longitudinal mortality follow-up utilized data from NHIS 2002 to 2014. NHIS is an annual, cross-sectional survey of US civilian non-institutionalized population conducted by the National Center for Health Statistics (NCHS) [26]. Each year NHIS samples near 35,000 households with estimated 87,500 persons with an oversampling of black, Hispanic, Asian and adults 65 years or older [26]. The NHIS sampling follows a multipurpose multistage area probability design; therefore data, when properly weighted, is representative of the US population [27]. A core set of questions is asked each year, and data can be pooled across years to enhance sample size [28]. The response rates for NHIS during the study period range from 73.8% to 89.6%. Participants aged 50 years and older were included in this investigation.

### Chronic conditions

Thirteen chronic conditions were included for the analysis: hypertension, coronary artery disease, other heart diseases, stroke, emphysema/COPD, asthma, cancer, diabetes, arthritis, kidney disease, hepatitis, depressive symptoms, and cognitive impairment. The conditions were selected based on the chronic condition list established by the US Department of Health and Human Services (DHHS) Initiative on Multiple Chronic Conditions (MCC) [29,30], which were available in NHIS. Participants were asked: Have you EVER been told by a doctor that you had… (i.e. hypertension, also called high blood pressure)? Coronary heart disease, angina pectoris, and myocardial infarction were placed into a category called “coronary artery diseases”. The kidney disease information was collected by asking “during the past 12 months, have you been told by a doctor that you had weak or failing kidneys?”.

Cognitive impairment was measured by the following question: “Are you limited in any way because of difficulty remembering or because experience periods of confusion?” Those who answered “yes” were categorized as “cognitively impaired”. This measure has been shown to produce cognitive impairment prevalence that are similar to estimates from using more precise case-ascertainment methods [31,32]. Depressive symptoms were assessed by using four of the six items from the Kessler-6 nonspecific psychological distress scale: “During the past 30 days how often do you feel (1) sad nothing could cheer you up, (2) hopeless, (3) that everything was an effort, (4) worthless?” [33,34] Responses were recorded on a 5 point scale ranging from 0 (none of the time) to 4 (all of the time) with a sum score above 8 considered having depressive symptoms [35]. The 8-point cutoff of the sum of 4 items is equivalent to the 12-point cutoff point for severe mental illness on sum of 6 items [33,35,36]

### Mortality linkage

NHIS data was linked with National Death Index (NDI) through probabilistic record matching using name, social security number, date of birth, state of residence, sex, race, and marital status. Vital status was obtained with date of death, and underlying causes of death if participants were deceased. We used the public-use linked mortality file which, for selected records, the date of death and/or underlying cause of death were subjected to data perturbation for privacy protection. The mortality follow-up was performed from the date of survey through December 31, 2015 [37]. 172,030 participants aged 50 years and older were involved in NHIS, of whom 166,126 (96.6%) had sufficient identification information to be linked with NDI for mortality follow-up. Our study examined all-cause mortality, and cause-specific mortalities due to cancer (ICD-10: C00-C97), heart diseases (I00-I09, I11, I13, I20-I51), cerebrovascular disease (I60-I69), chronic lower respiratory diseases (J40-J47), and Alzheimer’s Disease (G30).

The University of Miami Institutional review board approved this study and exempted the patient consent form requirement because the study was a secondary analysis of publicly available de-identified data.

### Covariates

The socio-demographic variables examined included age in years, sex (female vs. male), race (Black, Asian, other races vs. White), Hispanic background (vs. non-Hispanic), education level (high school graduate, above high school vs. less than high school), marital status (married vs. all other), health insurance (vs. no insurance), and household income (income to poverty threshold ratio < 1 (poor), ratio 1–1.99 (low income), and ratio > 4 (high income) vs. ratio 2–3.99 (middle class)). Health behavior variables included: smoking status (current, former vs. never), alcohol drinking status (none, moderate, heavy vs. light), and BMI (underweight, over-weight, obese vs. normal weight).

### Statistical analysis

A series of LCA models ranging from two to nine classes were estimated and compared. Model fit statistics included Akaike information criterion (AIC), Bayesian information criterion (BIC), sample-size adjusted BIC, for which smaller values of fit indices indicate a better model fit to the data. When a smallest value was not reached, a scree plot was utilized to select the optimal model. Entropy, an index summarizing the overall precision of the classification for all samples across classes, compared models. An entropy above 0.7 is considered a good value [38]. The Vuong-Lo-Mendell-Rubin and Lo-Mendell-Rubin likelihood ratio tests evaluated model improvement as the number of classes increased. The latent classes derived should have substantive clinical meaning and be distinct from each other. A parsimonious model is preferred when other indices are similar. LCA assumes that class indicators are conditionally independent of each other given class membership. We further examined the conditional independence with bivariate residuals (BVRs). Because BVRs are very sensitive to large sample size, we ordered the BVRs in descending order and allowed pairs with top bivariate residuals to correlate [39]. Participants were assigned to the class for which they had the highest probability of membership. Cross-tabulation of multimorbidity latent classes and socio-demographic and health behavior variables provided the characteristics of individuals in each class.

The multimorbidity latent class was used as a predictor of mortality. Cox proportional hazards models with the latent class as predictor and survival time as outcome were performed while controlling for covariates in the model. We examined the proportional hazard assumption by plotting the survival function over time. The survival curves that did not cross from each other indicating proportional hazard.

Introducing predictors or outcome variables to LCA models could potentially cause latent class shifts. We first estimated an unconditional LCA model and obtained the latent class and associated classification probabilities. During the regression steps, we adapted the manual three-step approaches [40] to minimize class shifts and adjust for potential bias when a latent class variable is used for prediction of distal outcome.

The LCA and survival analysis were performed using Mplus 8.0 (Muthén & Muthén). Descriptive statistics were calculated using SAS 9.4 (SAS Institute, Inc., Cary, NC) survey procedures. Analyses accounted for the complex survey design of NHIS with adjustments to the weight variable to account for the pooling of 13 years of data [27]. In the case of multiple comparisons, the α value and corresponding confidence intervals were Bonferroni corrected.

## Results

We utilized the study sample of 166,126 participants who had mortality follow-up information for all analyses. The study sample represents 89.2 million US adults aged 50 years and older. The average age of the sample was 64.9 years (SD 10.5) at the time of survey; 53.6% female, 82.9% White, 10.0% Black, 3.6% Asian, 3.5% other races. Hispanics made up 8.3% of the study population and 52.7% had above high school education. Of the 13 conditions included in the LCA, the prevalence of hypertension was 51.3%, highest, followed by arthritis (41.5%), and diabetes (16.6%).

### Identifying latent class chronic condition patterns in the population

**Fig 1:**
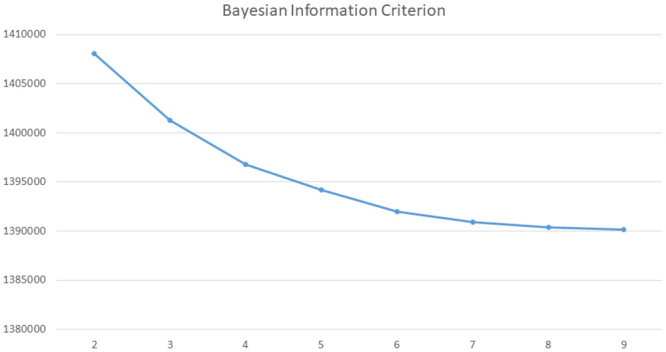
Scree plot of Bayesian information criteria (BIC) to select the optimal number of class model.

The model fit statistics improved as the number of classes increased, and a smallest value was not reached. Therefore, a scree plot was utilized to select the optimal number of classes (Fig 1). Minimum model improvement occurred beyond the five-class model, which had the highest entropy value of 0.64. The disease class identified by the 5-class model also represents clinically meaningful groups that share common underlying etiologies or risk factors. Therefore, the 5-class model was selected based on a combination of the model fit indices, parsimony, and clinical classification relevance. Upon examining the conditional independence of the 5-class model, we allowed the following pairs of conditions to correlate at the overall level—hypertension and diabetes, coronary artery disease and other heart diseases, coronary artery disease and stroke. We reached an improved 5-class model; the BIC value of the 5-class model with bivariate association is less than the BIC of both the 5 and 6 class models (Table 1).

**Table 1:** Model fit statistics for latent class analysis.

Number of latent class	Log Likelihood	Number of parameters	AIC*	BIC*	Adjusted BIC	Entropy
**2**	-703,854	27	1,407,763	1,408,033	1,407,948	0.603
**3**	-700,390	41	1,400,862	1,401,272	1,401,142	0.64
**4**	-698,057	55	1,396,225	1,396,776	1,396,602	0.57
**5**	-696,690	69	1,393,518	1,394,209	1,393,990	0.64
**6**	-695,475	83	1,391,116	1,391,948	1,391,684	0.628
**7**	-694,885	97	1,389,965	1,390,937	1,390,629	0.58
**8**	-694,539	111	1,389,301	1,390,414	1,390,061	0.596
**9**	-694,341	125	1,388,933	1,390,185	1,389,788	0.598
**5 with BVR association**	-695,250	72	1,390,645	1,391,367	1,391,138	0.64

*AIC—Akaike information criterion, BIC—Bayesian information criterion.

**Fig 2:**
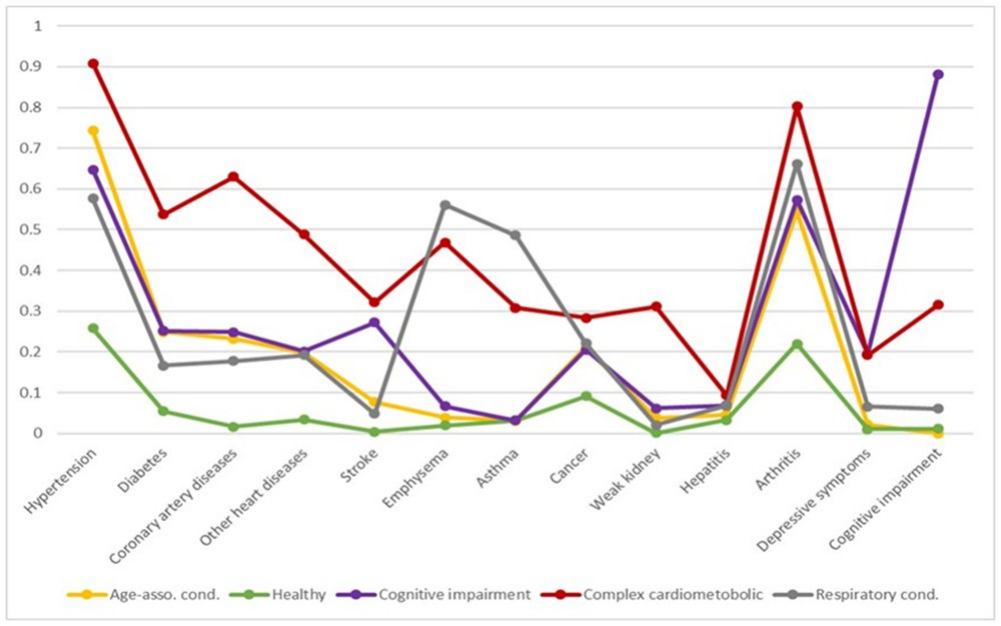
Probabilities of having chronic conditions for each latent class.

Classes were labeled according to their characteristic of the chronic conditions (Fig 2 and Table 2): 1) “Healthy” class (51.5% of the sample) is characterized by lower probabilities (<10%) of reporting any chronic condition except hypertension (26%) and arthritis (22%). Individuals in this class on average reported 0.7 conditions; 2) “Age-associated chronic conditions (hypertension & arthritis)” class (33.6%) is characterized by elevated risk of hypertension (74%) and arthritis (55%). Persons in this class had conditions that are often associated with aging and reported 2.8 conditions on average; 3) “Respiratory condition” class (7.3%) had high prevalence of emphysema/COPD (56%), asthma (49%) and arthritis (66%) and reported 3.7 conditions on average; 4) “Cognitively impaired” class (4.3%) displayed highly elevated risk of cognitive impairment (88%) and depressive symptoms (20%). This group also had high probability of having stroke (27%), hypertension (65%) and arthritis (57%) and 3.8 conditions on average; 5) “Complex cardiometabolic” class (3.2%) had the highest prevalence of hypertension (91%), diabetes (54%), coronary artery diseases (63%), other heart diseases (49%), stroke (32%), kidney disease (31%), cancer (28%) and arthritis (80%). On average, individuals in this group reported 6.5 conditions.

**Table 2:** Probabilities of reporting chronic condition for each latent class.

	Complex cardiometabolic	Cognitively impaired	Respiratory condition	Age-asso. cond.	Healthy
Hypertension	0.91	0.65	0.58	0.74	0.26
Diabetes	0.54	0.25	0.17	0.25	0.05
Coronary artery diseases	0.63	0.25	0.18	0.23	0.02
Other heart diseases	0.49	0.20	0.19	0.20	0.03
Stroke	0.32	0.27	0.05	0.08	0.00
Emphysema	0.47	0.07	0.56	0.04	0.02
Asthma	0.31	0.03	0.49	0.03	0.03
Cancer	0.28	0.21	0.22	0.21	0.09
Weak kidney	0.31	0.06	0.02	0.04	0.00
Hepatitis	0.09	0.07	0.07	0.05	0.03
Arthritis	0.80	0.57	0.66	0.55	0.22
Depressive symptoms	0.19	0.20	0.07	0.02	0.01
Cognitive impairment	0.32	0.88	0.06	0.00	0.01
Mean number of cond.	6.5	3.8	3.7	2.8	0.7
SD	1.2	1.3	1.1	0.9	0.6
N	5,304	7,223	12,163	55,853	85,583
%	3.2%	4.3%	7.3%	33.6%	51.5%

The socio-demographic and health behavior factors distributed differently across the five latent classes as displayed in Table 3. The “healthy” class is younger (average age of 62.0 years) and better educated (58.1% above high school education), while the “complex cardiometabolic”, the “age associated condition” and the “cognitively-impaired” class were older (average age 68.0, 68.1 and 70.1); and the “complex cardiometabolic” and “cognitively-impaired” class were less educated (36.0% above high school education). The “respiratory condition” class had a high percentage of females (65.5%). The “healthy” and the “age-associated condition” class had a higher percentage of married individuals (66.4% and 61.0%); whereas only 41.2% in the “cognitively impaired” class were married. The “complex cardiometabolic” class had the highest percentage of obesity (46.2%) and former smokers (44.5%).

**Table 3:** Distribution of demographic and health behavior variables by multimorbidity latent class*.

	Complex cardiometabolic (%)	Cognitively impaired (%)	Respiratory condition (%)	Age asso. conditions (%)	Healthy (%)	Total (%)
N	5,304	7,223	12,163	55,853	85,583	166,126
Age—Mean (SD)	68.0 (10.3)	70.1 (12.0)	65.7 (10.0)	68.1 (10.2)	62.0 (9.8)	64.9 (10.5)
Gender—Male	44.3	43.7	34.5	48.2	47.2	46.4
Female	55.7	56.3	65.5	51.8	52.8	53.6
Race—White	78.7	77.8	86.4	82.5	83.3	82.9
Black	14.3	14.6	9.4	11.5	8.6	10.0
Asian	2.3	3.0	1.4	2.8	4.5	3.6
All other races	4.6	4.6	2.8	3.2	3.6	3.5
Ethnicity-Hispanic	8.4	9.6	5.3	7.4	9.2	8.3
Non-Hispanic	91.6	90.7	94.7	92.6	90.8	91.7
Education < 12th grade	34.4	32.7	22.4	19.4	13.4	17.3
12th grade	30.0	31.3	31.9	32.1	28.5	30.1
Above 12th grade	36.0	36.0	45.8	48.5	58.1	52.7
Marital status-Married	47.4	41.2	54.9	61.0	66.4	62.3
All other	52.6	58.8	46.0	39.0	33.6	37.7
Had health insurance	96.0	94.9	93.5	94.9	90.0	92.2
No insurance	4.0	5.1	6.5	5.1	10.0	7.8
Income to poverty threshold ratio <1	25.8	23.7	15.2	10.1	7.1	9.8
Ratio 1–1.99	31.3	28.8	22.6	20.2	13.5	17.4
Ratio 2–3.99	28.4	29.3	31.8	31.8	28.0	29.5
Ratio 4 or +	14.5	18.3	30.4	37.9	51.5	43.3
Smoking—Never smoker	33.6	48.0	35.1	49.0	55.0	50.8
Current Smoker	21.9	17.2	24.2	12.5	16.0	15.7
Former Smoker	44.5	34.8	40.7	38.5	29.0	33.6
Drinking- Abstainer	68.5	65.2	49.3	48.0	36.6	43.2
Light drinker	24.5	24.1	36.0	35.1	41.7	38.0
Moderate drinker	5.0	7.4	9.7	12.7	16.4	14.1
Heavy drinker	2.1	3.3	5.0	4.2	5.3	4.7
BMI—Underweight	2.2	3.9	2.9	1.1	1.5	1.5
Normal weight	20.2	36.8	26.3	24.0	37.3	31.7
Overweight	31.4	31.3	31.1	37.6	39.8	37.9
Obese	46.2	28.0	39.7	37.2	21.4	28.8

*All p values of χ2 test are < 0.001. Percentages may not appear to add up to 100% due to rounding.

### The association between multimorbidity class and mortality

#### a) All-cause mortality

**Fig 3:**
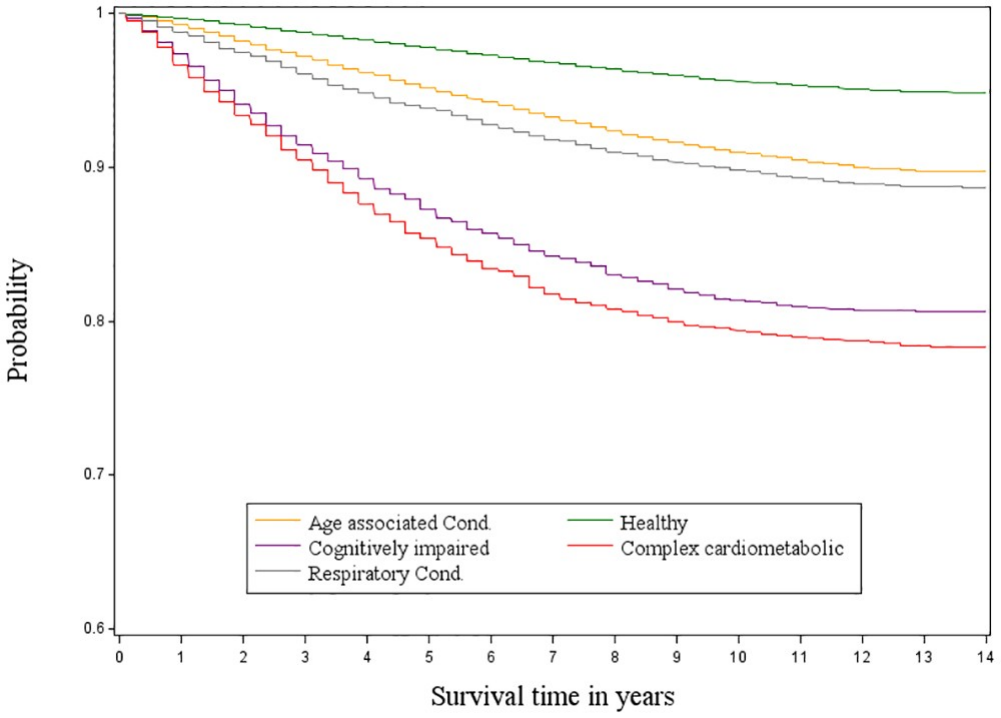
Kaplan-Meier survival curves of latent classes for all-cause mortality.

The average mortality follow-up time was 6.49 years (range <1 to 14 years); 28,129 (16.9%) deaths were observed. Cox proportional hazard model adjusting for covariates indicated participants in different multimorbidity classes had various elevated mortality risks compared to the “healthy” class. The “complex cardiometabolic” class had the highest mortality with a hazard ratio (HR) of 5.30 and a 99.5% confidence interval (CI) of [4.52, 6.22]; followed by the “cognitively impaired” class (HR 3.34 [2.93, 3.81]); the “respiratory condition” class (2.14 [1.87, 2.46]); and the “age-associated chronic condition” class (1.81 [1.66, 1.98]). (All P<0.001). (Table 4, Fig 3) The α and confidence intervals were Bonferroni corrected for 10 comparisons (0.05/10 = 0.005).

**Table 4:** Hazard ratios of all-cause mortality controlled for covariates*.

Multi-morbidity classes	Hazard Ratio	99.5% Confidence Interval	P value
Healthy	1.00		
Age-associated conditions (1)	1.81	[1.66, 1.98]	<0.001
Respiratory conditions (2)	2.14	[1.87, 2.46]	<0.001
Cognitively impaired (3)	3.34	[2.93, 3.81]	<0.001
Complex cardiometabolic (4)	5.30	[4.52, 6.22]	<0.001
C-cardiometabolic (4) vs. Cognitive impaired (3)	1.59	[1.27, 1.98]	<0.001
C-cardiometabolic (4) vs. Respiratory (2)	2.48	[1.98, 3.10]	<0.001
C-cardiometabolic (4) vs. Age asso. (1)	2.93	[2.46, 3.49]	<0.001
Cognitive impaired (3) vs. Respiratory (2)	1.56	[1.32, 1.85]	<0.001
Cognitive impaired (3) vs. Age asso. (1)	1.85	[1.63, 2.09]	<0.001
Respiratory cond. (2) vs. Age asso. (1)	1.18	[1.03, 1.36]	0.001

* Model controlled for age, sex, race, education, Hispanic ethnicity, marital status, household income, smoking status, drinking status, health insurance and BMI category.

Pairwise comparisons of mortality risk among the multimorbidity classes also showed statistically significant differences. The morality risk of the “complex cardiometabolic” class was 1.59 times higher than the “cognitively impaired” class (HR 1.59, 99.5% CI [1.27, 1.98], “complex cardiometabolic” vs. “cognitive impaired”, P <.001). The “cognitively impaired” class had a 56% higher risk of dying than the “respiratory condition” class (HR 1.56, 99.5% CI [1.32, 1.85], “Cognitively impaired” vs. “Respiratory condition”, P < 0.001). (Table 4).

#### b) Cause-specific mortalities (Table 5)

**Table 5:** Hazard ratios of cause-specific mortalities for each multimorbidity class*.

Multi-morbidity Class	Heart diseases death	Cerebrovascular disease death	Chronic lower respiratory disease death	Alzheimer’s disease death	Cancer death
	Hazard ratio	Hazard ratio	Hazard ratio	Hazard ratio	Hazard ratio
	95% CI	95% CI	95% CI	95% CI	95% CI
	P value	P value	P value	P value	P value
**No. of Deaths**	4754	1429	1642	773	6996
**Healthy group**	1.00	1.00	1.00	1.00	1.00
**Complex cardio-metabolic**	7.41	4.94	8.34	1.62	2.23
	[6.06, 9.05]	[3.23, 7.57]	[6.10, 11.39]	[0.81, 3.24]	[1.82, 2.74]
	<.001	<.001	<.001	0.17	<.001
**Cognitively impaired**	3.58	4.66	0.90	6.27	1.89
	[2.86, 4.48]	[3.42, 6.35]	[0.37, 2.18]	[4.74, 8.28]	[1.59, 2.26]
	<.001	<.001	0.82	<.001	<.001
**Age-associated conditions**	2.88	2.26	0.50	0.48	1.58
	[2.46, 3.37]	[1.69, 3.02]	[0.29, 0.86]	[0.31, 0.76]	[1.41, 1.78]
	<.001	<.001	0.012	0.002	<.001
**Respiratory condition**	1.20	0.96	13.65	0.08	1.87
	[0.82, 1.75]	[0.45, 2.05]	[10.82, 17.24]	[0.00, 7.00]	[1.59, 2.19]
	0.35	0.92	<.001	0.27	<.001

*Models adjusted for age, sex, race, Hispanic ethnicity, education, income, health insurance, smoking status, alcohol drinking status and BMI.

Cox proportional hazard model adjusting for study design and covariates indicated **heart disease** mortality was drastically elevated in the “complex cardiometabolic” class (HR 7.41, 95% CI [6.06, 9.05], P<0.001) compared to “healthy” class. The “cognitively impaired” class (HR 3.58 [2.86, 4.48], P<0.001) and the “age-associated condition” class (HR 2.88 [2.46, 3.37], P<0.001) were also associated with increased risk for heart disease death but not the “respiratory-arthritis” class (HR 1.20 [0.82, 1.75], P = 0.35).

The “complex cardiometabolic” class (HR 4.94 [3.23,7.57]), the “cognitively impaired” class (HR 4.66 [3.42, 6.53]), and the “age-associated condition (hypertension-arthritis)” class (HR 2.26 [1.69, 3.02]) all had heightened **cerebrovascular disease** mortality risk compared to “healthy” class (all P<0.001). The “respiratory condition” class did not show an increased cerebrovascular mortality risk (HR 0.96 [0.45, 2.05], P = 0.92).

The “respiratory condition” class (HR 13.65 [10.82, 17.24]) and the “complex cardiometabolic” (HR 8.34 [6.10, 11.39]) had substantially increased **chronic lower respiratory disease** mortality risk compared to the “healthy” class. The “age associated condition” class (HR 0.50 [0.29, 0.86], P<0.001) and the “cognitively impaired” class (HR 0.90 [0.37,2.18], P = 0.82) did not show increased respiratory disease mortality.

The “cognitively impaired” class had elevated risk of **Alzheimer’s Disease** death (HR 6.27 [4.74, 8.28], P< 0.001). The other three disease classes did not have elevated risk of Alzheimer’s disease death compared to the “healthy” class.

All four multimorbidity classes were associated with an increased risk for **cancer** mortality: “complex cardiometabolic” class (HR 2.23 [1.82, 2.74]), “cognitively impaired” class (HR 1.89 [1.59, 2.26]), “age-associated condition” class (HR 1.58 [1.41, 1.78]) “respiratory condition” class (HR 1.87 [1.59, 2.19]), and compared to “healthy” class (all P<0.001).

## Discussion

Our study is the first to investigate the relationship between the multimorbidity combination types and mortality in the U.S. older adult population using an innovative LCA approach. LCA reduced the complexity of data and identified five distinct meaningful subgroups in older adults: “healthy,” “age-associated chronic conditions,” “respiratory condition,” “cognitively impaired,” and “complex cardiometabolic.” We found four multimorbidity classes had various levels of elevated mortality risk, and the patterns of multimorbidity classes were strongly associated with the primary underlying cause of death. Additionally, the number of chronic conditions older adults experienced were not always proportional to the mortality risk they suffer.

Other studies that investigated the relationship between multimorbidity and mortality in older adults have measured multimorbidity as merely a cumulative count of conditions; these studies found dose-response positive relationships between numbers of conditions and the increased mortality risk [11,12,14]. Our study goes beyond measuring multi-morbidity as a count of conditions or arbitrary grouping. Instead, we employed the data driven LCA methodology and characterize the population into qualitatively distinct groups based on the relationship among the conditions.

Numbers of conditions is a straightforward and useful way of measuring multimorbidity. however, it weighs the impact of conditions equally. As our results show, groups reporting similar number of conditions can have significantly different mortality rates depending on the combinations of conditions. The “cognitively impaired” class reported on average 3.8 conditions, which was similar to the “respiratory condition” class who reported 3.7 conditions on average; however, the “cognitively impaired” class had a 56% higher all-causes mortality risk than the “respiratory condition” class (HR 1.56 [1.32, 1.85], P<0.001). These findings suggest certain chronic condition combinations such as cognitive impairment and depressive symptoms, had a substantial detrimental effect on overall morality in older adults.

The “cognitively impaired” class had an 88% chance of having cognitive impairment which is considerably higher than other classes. This class also had a 20% chance of reporting depressive symptoms and 27% of stroke. Cognitive impairment has been shown to be an independent predictor of mortality in older adults ever after controlling for multiple confounders [41]. Increased mortality was found even for very mild level of cognitive impairment [42]. Depression is associated with increased mortality in older persons and is a risk factor for Alzheimer’s Disease (AD) [43]. The prevalence of multimorbidity in older adults exceeds 60% [7]. A single cause of death may not capture the full range of diseases that older adults endured at the end of life. Our data show the conditions that frequently co-occurred with cognitive-impairment and depressive symptom includes stroke, cancer, arthritis and hypertension. The physiological health of the “cognitively impaired-depressive symptoms” class were not as severe as indicated by the relatively moderate number of conditions reported (3.8). However, cognitive impairment and depressive symptoms are debilitating conditions that affect the daily life of older adults and can cause increased disability and dependency on others [44,45], and prevented them from seeking proper medical care and adhering to proper disease management [46,47]. These could exacerbate older adults’ health situation and increase mortality risk.

The “complex cardiometabolic” class was not the oldest group identified in the study; the average age was 68.0 compared to 68.1 of “age associated condition” and 70.1 of “cognitively impaired”. However, this group had the highest mortality risk. The risk of dying in the “complex cardiometabolic” class was almost 3 time higher than the “age associated condition” class (HR 2.93, P<0.001) and 59% higher than the “cognitively impaired” class (HR 1.59, P < 0.001). In addition to having the highest prevalence for 9 out of the 13 chronic conditions, the “complex cardiometabolic” class was also the least educated, had the lowest income, and was most likely to be obese or former smoker among all classes (Table 3). These socio-economic disadvantages and poor lifestyle factors may have synergized with multiple chronic conditions and led to the highly increased mortality risk of the “complex cardiometabolic” class.

It was challenging to come up with names that summarized the condition of each multimorbidity class while still being succinct. For example, arthritis and hypertension were the two most common conditions in this age group with high prevalence in all four multimorbidity classes. We did not feel it was informative to include arthritis and hypertension in the name of all disease classes. Instead, we aimed to highlight the conditions that are not only most prevalent in a class but also distinct from other classes. However, the fact that arthritis and hypertension were a component of many of the disease classes, raises the possibility that underlying cerebrovascular disease or inflammatory response may relate to comorbid conditions and is an area of future research.

Our analysis of the cause-specific mortality demonstrated the patterns of multimorbidity classes were strongly associated with the primary underlying cause of death. The “complex cardiometabolic” class had the highest risk of heart disease death; and the “respiratory condition” class had the highest risk of chronic lower respiratory disease death. The “cognitive impaired” class had the highest risk of Alzheimer’s Disease mortality. Both the “cognitive-impaired” and the “complex cardiometabolic” classes had the highest risk of cerebrovascular disease death. This alignment of the primary underlying cause of death in each multimorbidity class confirms the categorization and naming of our multi-morbidity latent classes.

Although the percent of participants who were ineligible for mortality follow-up was very low (3.4%), we conducted analyses to determine if selection bias occurred during mortality follow-up, specifically whether the mortality follow-up ineligibility influenced one multi-morbidity class more than the other. The percentage follow-up ineligibility was the highest in the “healthy” class (4.2%), and lowest in the “complex cardiometabolic” class (1.8%) with the other three classes in the middle. Given the large sample size of the “healthy” class (over 88,500) and its health condition homogeneity (low prevalence in most conditions), the influence of mortality follow-up ineligibility bias was likely minimal. Therefore, for consistence reason, we utilized the study samples (96.6%, n = 166,126) who had mortality follow-up information for both the LCA analysis and the survival analysis.

One weakness of using LCA to study the multimorbidity in older adult population is the precision of the classification. The entropy which measures the precision of the classification of LCA models was only 0.64 and did not reach the ideal values of above 0.7. In contrast to our previous study which used NHIS data to examine multimorbidity patterns in the US general adult (age ≥18) population [48], an entropy value of 0.72 was achieved. Such reduction in classification precision have been reported [22] and is likely due to more heterogeneity in chronic condition patterns among older adults. This may be analogous to the phenomenon of reduced predictive utility of traditional risk factors in the older population. [49]. Other limitations include NHIS data were self-reported and subjective to recall bias. Only 14 of the 20 chronic conditions in the DHHS list [29,30] were available in NHIS data (“congestive heart failure” and “cardiac arrhythmias” were combined in “any other heart conditions” thus 14 instead of 13). The conditions that were not available in NHIS including autism, HIV, osteoporosis, schizophrenia, and substance abuse. Hyperlipidemia was only collected in some years of NHIS. Some of these conditions, such as autism, are not prevalent in the older population. We believe our study included the most important chronic conditions that have a strong influence on older adult mortality. The reference periods for the 14 chronic conditions were not consistent in NHIS; however, all conditions are chronic in nature. For example, even though cognitive impairment questions were asking about current state, cognitive impairment in older adult is generally a slow changing process, we believe the influence of the reference period on our study results is likely small.

Our investigation represents the first study to use national representative community-based data to study the pattern of multimorbidity combination type and mortality in the national level of US older adult population. The strength of the study includes large sample size, national representative data, and newly updated mortality linkage. It is expensive and difficult to evaluate a national sample by using standard validated cognitive-impairment ascertainment in a large population. The fact that the NHIS collects data from a representative sample of the US population annually with data linked to national death index makes it a powerful surveillance tool to study multimorbidity and mortality.

## Conclusions

Our study contributes to a better understanding of the effect of multimorbidity on the mortality in the US older adult population. Our findings provide valuable information for identifying vulnerable sub-populations such as the “complex cardiometabolic” and the “cognitively impaired” class who were at significantly increased risk of mortality. The results support an integrated and coordinated care system for older adults with multimorbidity to facilitate early intervention to prevent and treat related conditions. Our research suggests that primary care and geriatric physicians should pay close attention to signs of cognitive impairment and depressive symptoms in older patients. Even though they may not seem physiologically ill, these older adults face significantly heightened risk of mortality.
